# Identifying metrics of success for transitional care practices in childhood cancer survivorship: A qualitative interview study of parents

**DOI:** 10.1002/cam4.4164

**Published:** 2021-08-06

**Authors:** Karim Thomas Sadak, Milki Gemeda, Michelle C. Grafelman, Taiwo O. Aremu, Joseph P. Neglia, David R. Freyer, Eileen Harwood, Jude Mikal

**Affiliations:** ^1^ University of Minnesota Masonic Cancer Center University of Minnesota Masonic Children’s Hospital Minneapolis Minnesota USA; ^2^ University of Minnesota Minneapolis Minnesota USA; ^3^ University of Minnesota Medical School Minneapolis Minnesota USA; ^4^ Children's Center for Cancer and Blood Diseases Children's Hospital Los Angeles Los Angeles California USA; ^5^ USC Norris Comprehensive Cancer Center Los Angeles California USA; ^6^ Departments of Pediatrics and Medicine Keck School of Medicine University of Southern California Los Angeles California USA; ^7^ Division of Epidemiology & Community Health School of Public Health University of Minnesota Minneapolis Minnesota USA; ^8^ Division of Health Policy and Management School of Public Health University of Minnesota Minneapolis Minnesota USA

**Keywords:** Childhood cancer survivor transition qualitative parent

## Abstract

**Background:**

Survivor‐focused care for adolescent and young adult (AYA) childhood cancer survivors (CCS) often involves their parents. Recognizing the importance of parents in the ongoing care of CCS, our study sought to identify key aspects of a successful transition for CCS from pediatric‐ to adult‐centered care from the parent perspective.

**Methods:**

We conducted qualitative interviews with 26 parents of CCS who were receiving care in the long‐term follow‐up (LTFU) clinic at a single institution. We used a semi‐structured interview protocol with the parents and conducted a thematic content analysis.

**Results:**

Using a constant comparison approach, data revealed three primary themes regarding parents’ perspectives toward ensuring a seamless transition from pediatric‐ to adult‐centered follow‐up care: (1) the transition needs to include seamless communication between all involved parties, (2) survivors need to demonstrate sufficient health care self‐efficacy in order to achieve a successful transition, and (3) the survivor‐focused care should include support for survivors’ overall well‐being, including financial and health insurance literacy.

**Conclusions:**

For parents of AYA CCS, the optimal pediatric to adult care transition model should include mechanisms that facilitate communication between parents, CCS, and survivor‐focused providers while also supporting self‐efficacy and financial literacy as it relates to health insurance.

## Graphical abstract

Survivor‐focused care for adolescent and young adult (AYA) childhood cancer survivors (CCS) often involves their parents. To parents, adolescent and young adult (AYA) childhood cancer survivors (CCS), the optimal pediatric to adult care transition model should include mechanisms that facilitate communication between parents, CCS, and survivor‐focused providers while also supporting self‐efficacy and financial literacy as it relates to health insurance.

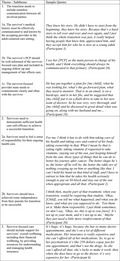

## 1 BACKGROUND

Childhood cancer diagnosis and subsequent treatment are traumatic for patients and their families. Parents of childhood cancer survivors may develop heightened concern for their child’s health and well‐being, which persists beyond cancer treatment and often results in overprotectiveness.^1^, ^2^, ^3^ As a result, many parents continue to accompany their older children to long‐term follow‐up (LTFU) appointments, as well as other medical visits.^4^, ^5^ Conversely, parents report that their presence at the follow‐up appointments provides survivors with companionship and support, strengthens their relationship with survivors, and fulfills their parental duty.^5^, ^6^ They have a strong sense of confidence in their abilities to communicate with health care providers because of their extensive experience as advocates for their children and their desire to continue aiding their adult children in management of their care.^5^, ^6^

Because parents continue to stay involved in survivors’ care and to see value in their continued investment even as survivors make the transition from pediatric‐ to adult‐centered LTFU, it is imperative to include parents in survivorship programs through appropriate mechanisms. Furthermore, research shows that harnessing parental engagement can benefit AYA CCS. A systematic review showed that parental involvement in diet and physical activity interventions was associated with better outcomes for childhood cancer survivors (CCS).^7^ Despite this, there is no evidence‐based standard practice for how to best engage parents in the LTFU care for their CCS. This includes how to leverage parental involvement to support the successful transition from pediatric‐ to adult‐centered care. The goal of our study was to identify parental drivers that promote a successful transition from pediatric‐ to adult‐centered survivor‐focused care.

## 2 METHODS

### 2.1 Study design

To identify indicators of a successful transition from pediatric‐ to adult‐centered survivor‐focused care from the perspective of parents of CCS, we conducted a phenomenological qualitative study, with parents of CCS as the key informants. Semi‐structured interviews were conducted with parents of CCS, and the transcribed interviews were subsequently analyzed for themes. This study design was previously used to conduct a similar inquiry of the medical providers involved in the care of CCS, as well as with adolescent and young adult CCS themselves. More study design details can be found in Sadak et al., 2017; 2020.^8^, ^9^

### 2.2 Participant recruitment

Following the Institutional Review Board (IRB) approval of this study (Study number: 1402E48101), parent participants were recruited during routine outpatient LTFU care appointments. Parents with children between the ages of 15 and 39 were introduced to the study by a medical provider at the conclusion of their visit. Then a clinical research assistant followed and either entered the patient room or found the parent in the clinic waiting room to provide an information sheet about the study, answer questions about the study from parents, and ultimately present the consent to be signed by participants. All participants had children who were current patients in the institution’s Childhood Cancer Survivor Program (CCSP), either in the pediatric or adult care unit. All patients continue to receive survivor‐focused care within the same CCSP. Care is delivered by pediatric oncology providers both in the pediatric and adult care settings. The model of care for survivors over the age of 25 years is adult‐centered and includes an internal medicine provider on the care team. According to their preference, parents were interviewed individually (one parent of the CCS) or as a pair (both parents of the CCS). Health information about the CCS was not collected in order to maintain the anonymity of the CCS and parents, though parents were asked to share their child’s diagnosis and age with the interviewer. Identifying information, including names of CCS and parents, were removed from the interview transcripts. Parents were ineligible only if they did not speak English or could not complete a phone interview due to a health condition or other reasons.

Per standard phenomenological qualitative study requirements, our goal was to interview at least 25 parents (or parent dyads) to achieve informational redundancy and content saturation.^10^ We approached a total of 30 parents, and 26 individuals or pairs ultimately participated in the interview process.

### 2.3 Data collection

Semi‐structured interviews were conducted with each of the participants or parent pairs. Each interview began with a review of the participant’s rights as research participants, and a review of key terms, such as “transition.” Transition, for the purposes of this study, was defined as the change from a pediatric‐centered clinic setting to an adult‐centered setting for childhood cancer survivor‐focused care.

Once participants had given their consent to participate in the study, interviews began with a single “grand tour” question,^11^ “Thinking about your child’s experience receiving care in the CCSP, how would you describe what you consider to be a successful transition?” This was followed by several probing questions to elicit more information about particularly meaningful points and to better understand the participants’ answers. The interviewer was trained to conduct this semi‐structured interview, and the lead author (KTS) reviewed interview transcripts throughout the process to provide feedback and revision based on the efficacy of the probing questions.

Interviews lasted 10–45 min in duration. No repeated interviews were necessary. Each interview was audio‐recorded then transcribed using a professional transcription service, Tybee Types. Identifying information, such as patient and parent names, were redacted from the transcripts prior to analysis. The interviews made up a total of 219 pages after redaction. The transcripts were uploaded to NVivo 9.0 for coding.

### 2.4 Analysis

Using NVivo 9.0 to read, organize, and code the interview transcripts, major themes were identified in the interviews by multiple authors (K.T.S. and M.C.G.). Initial codes were identified from a literature review^1^, ^2^, ^3^, ^4^, ^5^, ^6^, ^7^, ^8^, ^9^, ^12^, ^13^, ^14^, ^15^ and discussions between researchers. The coding researchers met at defined intervals to discuss the evolving themes within the pre‐determined categories, the associated quotes, and their interpretations and to refine the analysis approaches.

## 3 RESULTS

### 3.1 Participants

A total of 26 parents or parent pairs (interviewed simultaneously) participated in the study. All of them had children who were currently receiving care in our institution’s Childhood Cancer Survivor Program (CCSP) clinic and all were active participants in their child’s care. Survivors’ ages ranged from 16 to 38 years at the time of the interviews. All participants were from the Upper Midwest region of the United States. Information about the participants is detailed in Table 1.

**TABLE 1:** Descriptive information of interview participants and their children

Survivor diagnosis	Participant #	Mom/Dad/Both	Survivor sex	Survivor age (years)
CNS tumors				
Astrocytoma	25	Mom	M	31
Craniopharyngioma	16	Mom	M	32
Ependymoma grade III anaplastic	14	Mom	F	21
Germinoma	3	Mom	M	23
Medulloblastoma	7	Mom	M	37
	13	Mom	M	22
Solid tumors (non‐CNS)				
Bilateral retinoblastoma	9	Mom	M	22
Ewing's sarcoma	24	Mom	F	25
Nasopharyngeal carcinoma	1	Mom	M	22
Neuroblastoma	26	Both	M	16
Neuroblastoma multiforme	17	Mom	F	24
Orbital histiocytoma	10	Mom	F	38
Osteosarcoma	19	Mom	F	16
Leukemia/Lymphoma/Other				
Acute lymphoblastic leukemia	4	Mom	M	25
	5	Mom	F	32
	6	Mom	F	27
	12	Mom	F	24
	15	Mom	M	23
	18	Both	F	30
	21	Mom	M	21
	22	Mom	F	16
Acute myeloblastic leukemia	5	Mom	F	32
	20	Mom	M	28
Aplastic anemia	23	Mom	M	22
B‐cell lymphoblastic lymphoma	2	Mom	M	17
Hodgkin's lymphoma	11	Mom	F	27

### 3.2 Themes

Parent participants were asked to provide their perspectives of determinants of successful transitions for their CCS from pediatric‐ to adult‐centered care. From this information, three prominent themes emerged (detailed in Table 2): (1) the transition needs to include seamless communication between all involved parties, (2) survivors need to demonstrate sufficient health care self‐efficacy, and (3) survivor‐focused care should include support for survivors’ overall well‐being.

**TABLE 2:** Summary of themes and subthemes with key quotes

Themes/Subthemes	Key quotes
1. The transition needs to include seamless communication between all involved parties.	
1a. The survivor’s medical history must be effectively communicated to and known by the accepting provider in the adult‐centered care setting.	*They [the doctor] knew his story. He didn’t have to start from the beginning; they knew his story. Because that’s a hard story to tell over and over and over again, and I just think the whole transition was just, it really helped having people that knew him, appreciated him, and they accept him for who he is now as a young adult. (Participant 3)*
1b. The survivor’s PCP should be well‐informed of the survivor‐focused care plan and included in ongoing follow‐up and management of late effects care.	*I see her [PCP] as the main person in charge of his health, and I think everything should always be communicated to that primary. (Participant 26)*
1c. The survivor‐focused provider team needs to communicate clearly and often with the survivor.	*He has put together a plan for [my child], what he was looking for, what’s the go‐forward plan, what they need to monitor. That is in an email, is on a hardcopy, and is in her file, and he suggested that [my child] put it on a flash drive to take to her new team of doctors. So he was very, very thorough, and [my child] and he discussed in great detail what was going on, along with my husband and me… (Participant 24)*
2. Survivors need to demonstrate sufficient health care self‐efficacy to achieve a successful transition.	
2a. Survivors need to feel a sense of responsibility for their ongoing health care.	*For me, I think it has to do with him taking care of his health and taking care and control of his body, taking ownership in that. What I mean by that is eating right; taking vitamins if requested to take vitamins; staying out of the sun, just keeping himself from the sun; those types of things that he can do to better his journey after cancer. The better shape he’s in, the better off he will be, the better the odds are of nothing creeping up on him or anything like that. I can’t hold his hand on that kind of stuff, and I have to entrust to him that he takes his health seriously enough to put on 50‐block and stay out of the sun when appropriate and all that. (Participant 2)*
2b. Survivors should have achieved some independence from their parents for transition to be successful.	*I think then, maybe part of that treatment, when you transition, would be requiring that student, “Okay, [Child], you tell me what happened, and what you do know, and what are you supposed to do.” Test them on it. Make them responsible. I just think sometimes, we don’t say, “Okay, no, this is up to you [now]. It’s not up to your mom, and it’s not up to me.” Maybe they just need a little more reinforcement of that. (Participant 20)*
3. Survivor‐focused care should include support for survivors’ overall well‐being, including their financial well‐being, by providing resources for understanding and managing health insurance.	*It’s huge; it’s huge, because she has so many doctor appointments, and she’s on a lot of different medications. Her insurance is really expensive and she can’t afford all of her medications. So, to go to her psychiatrists it’s like 250 dollars copay just for one appointment, and that’s not the drugs. So she can’t afford all that; she’s lucky to make it work, then when she does have to go to the doctor, it’s very expensive for her. (Participant 6)*

Theme 1 The transition needs to include seamless communication between all involved parties.

According to the parent participants, communication is key to a successful transition for CCS. Communication needs to be effective and efficient between the pediatric and adult providers to ensure a smooth handover of care. Detailed communication also needs to occur between the survivor‐focused providers and the CCS’s primary care provider (PCP) to ensure they are well aware of the plan of care for the patient. The survivor‐focused care providers also need to communicate with the CCS about the transition process to ensure understanding and comfort in the transition.

Subtheme 1a The survivor’s medical history must be effectively communicated to and known by the accepting provider in the adult‐centered care setting.

Parents of CCS were clear that one of the foundations of a successful transition from pediatric‐ to adult‐centered care is communication between the team in the pediatric clinic and that in the adult clinic. Twenty‐five parents referred to the transfer of medical information between teams during their interviews. Additionally, 22 participants talked about the continuity of care, including several similar or overlapping comments about the transfer of knowledge about the CCS. Many of these parents relayed on stories of satisfaction and gratitude for their survivor‐focused care team knowing in‐depth information about their child, including past medical history as far back as their primary treating oncologist when they were first diagnosed. Parents expressed that they wanted that same level of detail to be passed on from the pediatric‐centered team to the adult‐centered team during the transition of survivorship care.

*Definitely sharing or sending over their records and their knowledge and the things they’ve learned would be very helpful, so questions don’t need to be asked on certain things; some things have already been covered. I would hope that his doctor at the pediatric clinic would share anything that might be concerning them with the new doctors. (Participant 4)*

Parents were comforted by the fact that their CCS is being cared for by a team who knows them well and understands their history. The participants reported that as their child becomes more independent and parent involvement in care is more limited, the presence of a care team that knows the CCS well makes granting that independence easier.

*I’m grateful that she has a team that she sees now on a regular basis, a follow‐up team to check her, to do her testing, and to check on her and make sure. Because before then, whenever anything would happen with her, our very first thought was oh my gosh, her cancer’s back. So we don’t ever want that to happen, but at least now, we know that she’s got doctors that are available to her, that can help her. (Participant 10)*

Parents similarly express a desire for their child to be known by the incoming adult‐centered providers. They also remarked that an excellent handover of information between the pediatric and adult care teams can reduce the burden of transition for CCS. Several participants reported that telling the childhood cancer story is challenging for the CCS, either because it forces the re‐emergence of traumatic memories or because the CCS was so young when they went through the treatments that they do not know the details well enough to recall easily. Therefore, a great weight is lifted by the pediatric‐focused medical team sharing the CCS’s medical history in detail with the adult‐focused care team. According to one participant,

*And [the doctor] knew his story. He didn’t have to start from the beginning; they knew his story. Because that’s a hard story to tell over and over and over again, and I just think the whole transition was just, it really helped having people that knew him, appreciated him, and they accept him for who he is now as a young adult. (Participant 3)*

Subtheme 1b The survivor’s medical history must be effectively communicated to and known by the accepting provider in the adult‐centered care setting.

During transition of care, PCPs often communicate frequently with pediatric oncologists. Twenty‐six parents expressed that this practice should be continued in adult care settings. However, parents’ opinions differed in the level of detail that should be given to the PCP. For example, one parent advocated for the PCP to know everything about the care of the CCS, including information about all of the specialists he/she visits, and the care provided by those specialists.

*I want her [PCP] to know anytime he goes to anybody. I see her as the main person in charge of his health, and I think everything should always be communicated to that primary. (Participant 26)*

On the other hand, another parent expressed an interest in the PCP being made aware of the CCS’s survivor‐focused care visits and transition but felt that the PCP did not need to know the details of that care.

*Theydon’t need to know every single thing, but just the major things, so I’ll just go back and pick information off to give them; I know that. (Participant 19)*

Despite these differences regarding the volume of detail provided to PCPs, the majority of parents agreed that having a well‐informed PCP who could handle issues outside of the survivorship clinic for the CCS is important.

*…maybe the family physician should get reports every year just so he’ll know, so if we would go in, he would have that information of what tests he just had and where he’s at so if there were any issues he would already know about it, wouldn’t have to request it and then wait for it to come back. (Participant 8)*

Subtheme 1c The survivor‐focused provider team needs to communicate clearly and often with the survivor.

Parents spoke often about the need for the medical team, both in the pediatric and adult clinics, to communicate directly with CCS, who at the time of transition are emerging young adults, learning to take on more responsibility and ownership of their health care. Therefore, pediatric providers, who spoke mostly with parents during the CCS’s cancer diagnosis and treatment period, need to explain the CCS’s history and follow‐up plan directly with their patient.

*He has put together a plan for [my child], what he was looking for, what’s the go‐forward plan, what they need to monitor. That is in an email, is on a hardcopy, and is in her file, and he suggested that [my child] put it on a flash drive to take to her new team of doctors. So he was very, very thorough, and [my child] and he discussed in great detail what was going on, along with my husband and me… (Participant 24)*

Twenty parents discussed the importance of the clinic having accurate contact information for the CCS, knowing that young adulthood is a time of frequent movement, so they can contact the CCS at any time with appointment or follow‐up reminders.

*They would have to maybe have a list of kids and know when the kid is graduating and contact them and their family maybe before so they can find out what their plans are, where they’re going, and then follow up to make sure that they ended up doing what they thought they were going to do and have that updated contact info.(Participant 26)*

Twenty parents also described the need for education and information regarding cancer and late effects, as well as the transition to the adult‐centered clinic. In particular, 20 parents reported desires for the pediatric clinic to provide preparation for the CCS, often involving specific instructions. These instructions should include when the CCS should follow‐up with the adult‐focused provider, what testing needs to be done at specific times, and where exactly the patient should go for the appointment at the adult‐centered clinic.

*…just really saying, 'This is what it'll look like when you go. They're going to ask you these questions, and it's going to be like this. It's not going to be how we do it, most likely.' Maybe they did say that a couple of times; I can't remember. But just to make sure that they are explaining those kinds of things. (Participant 19)*

Theme 2 Survivors need to demonstrate sufficient health care self‐efficacy in order to achieve a successful transition from the pediatric to the adult setting.

Many parent participants spoke about the need for their CCS to be ready for the transition before undertaking it, and a key component of their readiness is self‐efficacy. Specifically, the parents stated that CCS needed to exhibit agency, independence, and clear self‐efficacy before parents would see their CCS as ready to move into adult‐centered care settings. While many parents still wanted to be involved in their CCS’s care into their child’s young adulthood, they understood that transitioning from a pediatric‐centered clinic to an adult‐centered setting requires the CCS to be able to understand their own history and to carry out the necessary tasks to maintain their health.

Subtheme 2a Survivors need to feel a sense of responsibility for their ongoing health care.

Nineteen participants spoke of the need for their CCS to take responsibility for their health care. However, the meaning of responsibility varied among the parents. One father remarked that their child would benefit from going to further visits with her mother in order for the parent to pass on knowledge of the disease that the child does not remember because they were too young.

*I think in general, having specifically [Mom] with [Child] as she goes through some of these things now, allows [Mom] to hand off some of the thoughts that she had at the time, that [Child] was too young to understand, that now she can take ownership of in herself. (Participant 18)*

Other parents reported hopes that their child would take full responsibility by listening to instructions given by the medical team, engaging in health‐promoting behaviors, and following up regularly throughout the rest of their life.

*For me, I think it has to do with him taking care of his health and taking care and control of his body, taking ownership in that. What I mean by that is eating right; taking vitamins if requested to take vitamins; staying out of the sun, just keeping himself from the sun; those types of things that he can do to better his journey after cancer. The better shape he’s in, the better off he will be, the better the odds are of nothing creeping up on him or anything like that. I can’t hold his hand on that kind of stuff, and I have to entrust to him that he takes his health seriously enough to put on 50‐block and stay out of the sun when appropriate and all that. (Participant 2)*

Even though the definition of health care responsibility differed among the parent, many agreed that the CCS needed to “buy‐in” to the idea of LTFU. It is often difficult, they purported, to convince young adult CCS to continue following up in a LTFU clinic, especially as the CCS move further from their diagnosis and treatment periods. Parents wanted help from the LTFU clinic providers in educating CCS and convincing them of the importance of follow‐up.

*I suppose just stress to him that it is important for him to come back every year, because that’s what they told us, that he needs to have a check‐up every year and an MRI every other year, I don’t know, forever. So encouraging him to keep that part up. I could see, as he gets to be maybe 30s, middle age, all this is getting ridiculous. If the cancer doesn’t recur, then they’re going to think, ‘Well I don’t need to go to the doctor anymore.’ I can see where that would happen. So encouragement that he should keep coming and to monitor everything. (Participant 13)*

Subtheme 2b Survivors should have achieved some independence from their parents for transition to be successful.

Twenty parent participants reported increasing independence of the CCS, in terms of living situation and attending appointments on their own, is an indicator of a successful transition from the pediatric‐ to adult‐centered care setting.

*I think he’s very proactive, so I can see him stepping into the role of, even potentially, at some point, going to his appointments without Mom and Dad, or us meeting him down there for lunch after, or something like that. I can imagine that. (Participant 2)*

Part of the CCS’s independence needs to include adequate knowledge of their own medical history and LFTU needs. This knowledge enables CCS to attend appointments alone and relay on their history to providers without the need for parental input. One parent remarked that providers could help boost the CCS’s independence by encouraging them to personally know the details of their treatment and late effects monitoring.

*I think then, maybe part of that treatment, when you transition, would be requiring that student, “Okay, [Child], you tell me what happened, and what you do know, and what are you supposed to do.” Test them on it. Make them responsible. I just think sometimes, we don’t say, “Okay, no, this is up to you [now]. It’s not up to your mom, and it’s not up to me.” Maybe they just need a little more reinforcement of that. (Participant 20)*

Theme 3 Survivor‐focused care should include support for survivors’ overall well‐being, including their financial well‐being, by providing resources for understanding and managing health insurance.

Most parent participants (*n* = 23) commented that insurance coverage and management are a challenge for CCS as they transition into young adulthood and gain independence.

*It’s huge; it’s huge, because she has so many doctor appointments, and she’s on a lot of different medications. Her insurance is really expensive and she can’t afford all of her medications. So, to go to her psychiatrists it’s like 250 dollars copay just for one appointment, and that’s not the drugs. So she can’t afford all that; she’s lucky to make it work, then when she does have to go to the doctor, it’s very expensive for her. (Participant 6)*

In addition, since insurance plans often differ as survivors age out of coverage under their parents’ plan, the expenses related to follow‐up tests are shocking to survivors. Parents felt it was important LTFU clinic providers to have conversations with their children about the necessary frequency of various tests and accommodations for related financial difficulties.

*[Child] works full‐time, but it’s just under the hours, and it’s a small business, so he does not get health insurance. So he pays for that privately. He was under UCare, and that was a pretty good plan, but this plan now doesn’t cover his prescriptions or anything, and his deductible is like 2000. And [Dad] and I have paid for quite a bit of it. But his MRI, we talked to the doctors, and they said, ‘Okay, then why don’t we do it like every other year, every two years?’ So on those years, we do all his doctoring; we do the MRI, we see all of the various doctors, and we do the long‐term clinic. That way, he meets the deductible. And that’s been helpful, a little bit. But he drags his feet. ‘I don’t want to go to the doctor. It’s just going to be another bill for me.’ And I’m sure you’ve seen that. (Participant 16)*

Several parents remarked that having easily understood resources and educational messages available through the survivor‐focused care clinic to help CCS choose and manage their insurance options would be beneficial as parents had gained this knowledge over decades of experience navigating the complexity of health insurance coverage and their child needed to learn these same lessons on an accelerated timeline.

## 4 CONCLUSIONS

Parents remain an integral part in comprehensively caring for CCS, hence the need to identify, validate, and leverage their involvement and insights to improve the transition of care to adulthood. This study identified parent‐reported factors that may facilitate the transition of care for CCS. In ways that respect survivor autonomy, providers must consider parents' integral contribution to the care of their children and acknowledge their possibly dwindling involvement as CCS age and transition to adult‐centered care. Clinical programs that transition care for CCS may benefit from providing specialized approaches to address parental concerns raised through this work. From the parent perspective, the most important aspects determining the success of the transition from pediatric‐ to adult‐centered survivorship care are communication among the involved parties, self‐efficacy of the CCS, and the availability of resources to aid the CCS in managing the non‐medical components of their health care, such as health insurance.

Our findings echoed those of earlier studies that highlighted parental concerns about the transition of care and the loss of familiarity, comfort, and trust that they have established with their pediatric‐focused providers.^12^, ^13^ The loss of continuity with providers is challenging for parents and some CCS for several reasons, including a reliance on these providers to remember their child’s complex cancer history and evolving LTFU needs. As previously reported, CCS and parents also experience the re‐emergence of traumatic memories when recounting to new providers their child’s cancer diagnosis and treatments.^14^ While acknowledging the challenges that providers also encounter during the transition, parents asked that the CCS' detailed medical history always be given to the new adult care providers. This would greatly benefit both parent and survivor and eliminate some of the need to explain all of their medical history to new providers. This benefit has also been reported in previously published literature on this topic^12^ Similarly, to facilitate better care and clinical outcomes for their CCS, parents hoped that the survivor‐focused providers would also contact their child’s primary care provider as part of the transition‐related provider‐to‐provider communication.

Parents also mentioned that all survivor‐focused providers should promote a culture of open and direct communication. Because most CCS were much younger during their cancer therapies, most communication and information‐sharing initially occurred between providers and parents only. As CCS emerge as young adults and prepare to take ownership of their health care, providers need to progressively increase their communication of plans, recommendations, and follow‐up needs directly with the CCS. This narrative is consistent with prior studies that suggest AYA CCS are not getting the intended messages communicated by providers, as many may think that they only need to visit their LTFU clinics when they have symptoms.^15^ As a result, providers face the challenge of communicating clearly with the CCS to provide education regarding late effects and the necessity of LTFU and ultimately elicit buy‐in from the CCS.

While parents wanted clear instructions for their CCS, many parents also believe that increased independence of their CCS is essential for successful transitioning. Parents want to see their children taking ownership of their health and follow‐up care. In this study, parents described a need for providers to assess the CCS’ readiness for transition, a strategy that has previously been discussed as a means, in conjunction with the formulation of a transition plan, to help CCS increase their independence and responsibility.^12^ Lack of autonomy in CCS may result in reduced knowledge about their cancer history and current health needs, as well as decreased motivation to seek long‐term survivor‐focused care.^16^

Additionally, parents had major concerns about their AYA‐aged CCS’ abilities to appropriately choose health insurance plans and manage the costs associated with their health care. For many young adults, including those without the history of a childhood cancer or other chronic conditions, the task of understanding, choosing, and maintaining an insurance plan that is separate from their parents’ is overwhelming. In addition, because CCS often have other underlying conditions that may affect the type of coverage they are eligible for, financial challenges continue to pose a substantial barrier to LTFU care for AYA CCS.^12^ When financial toxicity ensues, the CCS' comprehensive well‐being, health‐related quality of life, and quality of care are adversely affected.^16^ Hence, parents would like the survivor‐focused care clinic to recognize the CCS’ knowledge gap and provide resources to help CCS manage these issues as part of a comprehensive care plan. To accomplish this goal, the care team could be expanded to include patient navigators and social workers tasked with delivering personalized insurance educational and vocational counseling that can help prevent insurance‐related financial toxicity.

Selection and participation biases were limitations of this study resulting from the recruitment of participants from a single institution that likely had favorable experiences with their child’s transition of care. The participants were almost all mothers leaving the father perspective mostly absent. These parent participants have also only been exposed to one model of survivor‐focused care and may not have the breadth of knowledge to know of other methods that may have improved their child’s transition. To combat this bias, we recruited parents with children of a variety of ages, ranging from adolescence to late young adulthood, some of whom had undergone transition already, and some who were still being seen in the pediatric‐centered setting. But for many young adult‐aged survivors, the mere presence of their parents may have suggested that the CCS were less independent and thus parental input reflected an additional bias of being more engaged in their child's survivor‐focused care since as parents they also attended the visit. In designing the study, we also considered the possibility of experimenter bias. To address this, multiple coders (K.T.S. and M.C.G.) participated in reading the transcripts and identifying themes. Other researchers periodically reviewed the themes, as well. Another limitation of the study emerged through reading of the interview transcripts as participants reported variations in the specifics of how they expected the transition to go. A few parents described greater involvement in their CCS’ life and care because the prior treatments resulted in physical or intellectual deficits that either delayed or made impossible that some CCS achieve full independence from their parents. This was most often described by parents of CCS who had suffered from CNS and non‐CNS cancers. This finding is consistent with previous reports where similar challenges in attaining complete independence by those who suffered childhood CNS and non‐CNS cancers were noted.^17^, ^18^, ^19^, ^20^ Because this group of parents was in the minority, their experience may have been under‐represented in the recurrent themes identified.

For an overwhelming majority of parents interviewed, the indicators of successful transition for their CCS from the pediatric‐ to adult‐centered survivor‐focused care setting were aligned and clear: (1) communication among all involved parties, including providers and patients, is essential, (2) CCS need to be relatively independent from their parents and take responsibility for their health, and (3) other resources, including those to help with insurance issues, need to be available to care for the overall well‐being of CCS.

## ETHICAL APPROVAL STATEMENT

This study was approved by an Institutional Review Board (Study number: 1402E48101) before its commencement.

## CONFLICT OF INTEREST

No conflict of interest reported by any of the authors.

## AUTHOR CONTRIBUTIONS

Karim Thomas Sadak designed the study, performed the analysis, and wrote the manuscript. Milki Gemeda performed the analysis and wrote the manuscript. Michelle C. Grafelman performed the analysis and wrote the manuscript. Taiwo Opeyemi Aremu reviewed/edited the manuscript. Joseph P. Neglia reviewed/edited the manuscript. David R. Freyer designed the study and reviewed/edited the manuscript. Eileen Harwood designed the study. Jude Mikel reviewed/edited the manuscript. All authors read and approved the final manuscript.

## Data Availability

The data that support the findings of this study are available upon request from the corresponding author. The data are not publicly available due to privacy or ethical restrictions.
